# Taxifolin synergizes Andrographolide-induced cell death by attenuation of autophagy and augmentation of caspase dependent and independent cell death in HeLa cells

**DOI:** 10.1371/journal.pone.0171325

**Published:** 2017-02-09

**Authors:** Mazen Alzaharna, Iyad Alqouqa, Hon-Yeung Cheung

**Affiliations:** Research Group for Bioactive Products, Department of Biomedical Sciences, City University of Hong Kong, Hong Kong SAR, China; National Institute of technology Rourkela, INDIA

## Abstract

Andrographolide (Andro) has emerged recently as a potential and effective anticancer agent with induction of apoptosis in some cancer cell lines while induction of G2/M arrest with weak apoptosis in others. Few studies have proved that Andro is also effective in combination therapy. The flavonoid Taxifolin (Taxi) has showed anti-oxidant and antiproliferative effects against different cancer cells. Therefore, the present study investigated the cytotoxic effects of Andro alone or in combination with Taxi on HeLa cells. The combination of Andro with Taxi was synergistic at all tested concentrations and combination ratios. Andro alone induced caspase-dependent apoptosis which was enhanced by the combination with Taxi and attenuated partly by using Z-Vad-Fmk. Andro induced a protective reactive oxygen species (ROS)-dependent autophagy which was attenuated by Taxi. The activation of p53 was involved in Andro-induced autophagy where the use of Taxi or pifithrin-α (PFT-α) decreased it while the activation of JNK was involved in the cell death of HeLa cells but not in the induction of autophagy. The mitochondrial outer-membrane permeabilization (MOMP) plays an important role in Andro-induced cell death in HeLa cells. Andro alone increased the MOMP which was further increased in the case of combination. This led to the increase in AIF and cytochrome *c* release from mitochondria which consequently increased caspase-dependent and independent cell death. In conclusion, Andro induced a protective autophagy in HeLa cells which was reduced by Taxi and the cell death was increased by increasing the MOMP and subsequently the caspase-dependent and independent cell death.

## Introduction

Andro, a diterpenoid lactone, is the major bioactive constituent of the herb *Andrographis paniculata* and is mainly implicated towards its pharmacological activity [1]. Different studies have shown the various bioactivities of Andro including anti-inflammatory [2], anti-microbial [3], immunomodulatory [1], hepatoprotective and cardioprotective [4, 5]. Recently, many studies have shown Andro as an effective anticancer agent [1, 6–8]. It has different effects on different cancer cell lines depending on their physiological background and histological origins [9]. It causes apoptosis in MCF-7 and HL-60 [1, 6] while causes cell cycle arrest with weak apoptosis in HepG2, Hep3B and DU145 [10–12]. Andro was shown to induce apoptosis by increasing ROS generation and activation of p38, JNK and p53 in different cancer cells [10, 13, 14]. The combination of Andro with other drugs was also found to be effective and synergistic in the few studies done [12, 15, 16].

The traditional mono-target therapy protocol for cancer treatment is becoming increasingly ineffective and may lead cancer cells to develop acquired resistance due to the complexity of cancer and its signaling pathways [17]. Combination or multi-component therapy, in which one or more drugs are used at the same time, seems like a possible option [18]. This can be approached by combination of different mechanism-based agents or the development of multi-target molecules [19]. This alternative strategy will increase the efficiency of therapy and minimize toxicity. Dietary supplements and other phytotherapeutic agents that are chemically complex are an important starting materials for the discovery of newer synergistic combinations and single agent multi-target drugs [20].

Flavonoids, phenolic natural products, present abundantly in the plant kingdom [21, 22]. Many studies have presented the different biological effects of flavonoids including anticarcinogenic effects [21, 23]. The beneficial effects of flavonoids in cancer therapy have been attributed to different mechanisms [24, 25]. The flavonoid Taxi was shown to have anti-oxidant effect and also possess antiproliferative effects against different cancer cells [26–29]. Many studies have also pointed out the synergistic effects of flavonoids when used in combination with other compounds [30–32].

Recently, two research papers have indicated the effect of Andro on autophagy in different cancer cell lines [33, 34]. Autophagy is a catabolic process through which cellular systems maintain a homeostatic equilibrium [35]. Cancer cells use autophagy as a survival mechanism under unfavorable conditions like hypoxia, lack of nutrients or due to chemotherapy treatment where it leads to therapeutic resistance. Therefore, the inhibition of autophagy in these cases can improve the cytotoxic effects of the drug [36].

Although many researchers have investigated the effect of Andro on many types of cancer cell lines, a few studies have investigated the combined-effect of Andro with other compounds and especially with other natural compounds. Therefore, to our knowledge, this is the first study conducted to investigate the effect of Andro alone or in combination with Taxi on apoptosis and autophagy in HeLa cells.

## Materials and methods

### Cell culture and reagents

HeLa cells were kindly provided by Prof. WF Fong (City University of Hong Kong, Hong Kong SAR) while MCF-7 cells were obtained from ATCC (Manassas, VA, USA). The HeLa cells were tested and authenticated based on STR profiling using Promega PowerPlex^®^ 18D System and analyzed using the ABI 3130 Genetic Analyzer (DiaCor, Hong Kong SAR). The two cell lines were maintained in DMEM medium supplemented with 10% fetal bovine serum (Invitrogen, CA, USA), 100 units/ml penicillin and 100 μg/ml streptomycin (Sigma, MO, USA) at 37°C in a humidified 5% CO_2_ incubator. All other reagents were obtained from Sigma-Aldrich unless otherwise indicated.

### Bioactive compounds for cells treatment

The bioactive compounds used were: Andro (97%, HPLC) (Indofine Chemical Company, NJ, USA). Taxi (94%, HPLC) was obtained from Sigma, MO, USA.

### Primary and secondary antibodies

p-p53 (Ser15), p-JNK (Thr183/Tyr185), PARP, Caspase 3, Caspase 7, Caspase 9, LC3-II, Bcl-2, Bax, Bid and Bim were obtained from Cell Signaling Technology, Beverly, MA. AIF, Cytochrome *c*, Lamin A/C, CruzFlour (CFL) 647-conjugated anti-mouse, CFL 488-conjugated anti-rabbit and CFL 647-conjugated anti-rabbit were obtained from Santa Cruz Biotechnology, Santa Cruz, CA. GAPDH was obtained from Abcam, Cambridge, UK. β-actin was obtained from Sigma, MO, USA while Cytochrome *c* was obtained from Invitrogen, CA, USA).

### Cell viability using the MTT assay

Cell viability was assessed by using 3-(4, 5-dimethylthiazol-2-yl)-2, 5-diphenyl-tetrazolium bromide (MTT) (Invitrogen, CA, USA). Cells were seeded at 4000 cells/well into 96 wells plate, incubated in 5% CO_2_ at 37°C for 24 h and the next day the medium was removed and a medium containing drugs was added and the cells were incubated for the indicated period of time. At the end of the treatment, the medium was removed and a medium containing 0.5 mg/ml MTT was added to each well. The plate was then incubated for an additional 4 h. The supernatant was removed at the end of incubation and 100 μl DMSO were added to each well to dissolve the formazan crystals that had formed and the plate was left for 30 minutes. The absorbance was measured at 570 nm using a multi-well scanning spectrophotometer (PowerWave HT, BioTek, USA). Cell viability was expressed as percentage of control by comparing the number of live cells in the treated group to the number in the vehicle control group. CompuSyn software (CompoSyn, Inc., NJ, USA) was used to calculate IC_50_, Combination index (CI) and The Dose Reduction Index (DRI). (The CI > 1 indicates antagonism, CI = 1 indicates additive and CI < 1 indicates synergism.

### Apoptotic cell death assay

For quantification of apoptotic cells, the Yo-Pro-1 assay kit (Invitrogen, CA, USA) was used and the procedure provided by the manufacturer was followed. Briefly, the cells were seeded in 60 mm plates and drugs were added after incubation of cells for 24 h. The cells were left incubated for the designated time and then detached from the plate using trypsin, 1x10^6^ cells were collected, washed with cold PBS and then resuspended in 1 ml PBS. Then, 1 μL of Yo-Pro-1 solution and 1 μL of propidium iodide (PI) solution were added to the cell suspension. Cells were incubated for 30 min. on ice in the dark and then the fluorescence results were read using the flow cytometer (Becton Dickinson, CA, USA) and analyzed by CellQuest Software (Becton Dickinson, CA, USA). Where the viable cells are Yo-Pro-1 ^−^ /PI ^−^, early apoptotic cells are Yo-Pro-1^+^/PI ^−^ and the late apoptotic or primary necrotic cells are Yo-Pro-1^+^/PI ^+^.

### Quantification and detection of autophagic vacuoles

For quantification of cells with Acidic Vesicular Organelles (AVOs), the cells were seeded in 60 mm plates and drugs were added after incubation of cells for 24 h. The cells were left incubated for the designated time. Acridine orange was then added to the plates where the final concentration was 1 μg/ml and the cells were incubated for an additional 15 min. Then cells were detached from the plate using trypsin, washed with cold PBS, resuspended in 1 ml PBS and AVOs were quantified using the flow cytometer and the results analyzed using CellQuest software. The red fluorescence of the cells increases as the number of AVOs increase. On the other hand, the AVOs were detected in the cells by using Cyto-ID Autophagy Detection Kit (Enzo Life Sciences, France). The Cyto-ID Green detection reagent serves as a selective marker of autophagosomes and autolysosomes. The protocol provided by the manufacturer was followed. Briefly, the cells were grown on sterile coverslips and the drugs were added after 24 h of incubation. The cells were left incubated for the designated time, and then the medium was removed, cells were washed twice with assay buffer. Then 100 μl of assay buffer containing Cyto-ID Green detection reagent (2 μl/ml) and Hoechst 33342 (1 μl/ml) were added and cells incubated for 30 min. Later, the cells were washed with 100 μl assay buffer, mounted on microscope slides and analyzed using the Leica TCS SPE confocal microscope (Leica Microsystems, Wetzlar, Germany).

### Transmission Electron Microscopy (TEM) analysis

Cells were seeded in a 35 mm plate containing thermanox coverslips, treated with DMSO or 50 μM Andro for 24 h. The protocol used was modified from Cheung *et al*. (2012). Briefly, cells were fixed in primary fixative (2.5% glutaraldehyde, 2% paraformaldehyde in 0.1 M Sorenson's Buffer) (EMS, Hatfield, USA). Cells were rinsed with 0.1 M Sorenson's Buffer. Then fixed in a secondary fixative (2% osimium tetroxide) (EMS, Hatfield, USA), then cells were dehydrated and embedded. The blocks were trimmed and sectioned and after staining with uranyl acetate and Reynolds lead citrate (EMS, Hatfield, USA), the sections were imaged on an electron microscope (Philips Electronics, Netherlands).

### Measurement of ROS

The cells were seeded into 60 mm plates, treated and incubated for the designated time. The 2′,7′-dichorofluorescein diacetate (DCF-DA) was added to the cells (10 μM) in the last 30 min of drug exposure. DCF-DA is de-esterified intracellularly and turns to highly fluorescent compound (DCF) upon oxidation. After the designated time of incubation, the cells were harvested, washed and resuspended in ice-cold PBS. PI (5μg/ml) was added for gating the viable cells and the cells were incubated for another 10 min. The DCF fluorescence was then determined by the flow cytometer and the results were analyzed using the CellQuest Software.

### JC-1 Staining

The cells were seeded into 60 mm plates, treated and incubated for the designated time. Then the cells were harvested, washed and resuspended in PBS. The cells were then labeled with the JC-1 reagent (4 μM) (Invitrogen, CA, USA) for 15 min at 37°C. After washing, cells were resuspended in PBS and the fluorescence was measured using the flow cytometer and the results were analyzed using the CellQuest Software. The stained cells were observed by growing the cells on coverslips, treatment for the desired time and then staining using JC-1 for 30 min. The coverslips were then mounted on microscope slides and the slides observed using the fluorescence microscope (Zeiss Axioskop, Mikron Instruments, NY, USA). JC-1 enters into mitochondria and reversibly change color from green to red as the membrane potential increases. In healthy cells with high mitochondrial membrane potential (MMP), JC-1 spontaneously forms complexes known as J-aggregates with intense red fluorescence. On the other hand, in apoptotic or unhealthy cells with low MMP, JC-1 remains in the monomeric form, which shows only green fluorescence.

### Protein lysate of cytoplasm and nucleus

Nuclear and cytoplasmic lysates were prepared according to the protocol from the company (Cayman Chemical, Ann Arbor, MI, USA). Briefly, cells were seeded in 100 mm plates and treated with the specified compounds after 24 h and left for the specified time. Cells were collected using scrapper, counted, washed 2 times with cold PBS containing phosphatase inhibitors and then ice-cold hypotonic buffer was added to the pelleted cells. Nonidet P-40 was added, tubes were centrifuged briefly and supernatant was collected (cytoplasmic fraction). Then the pelleted nuclei were lysed using ice-cold extraction buffer containing a mixture of protease and phosphatase inhibitors. Tubes were then centrifuged at 14000 x g for 10 minutes at 4°C and the supernatant was collected (nuclear fraction). Then the protein concentration was measured and the lysates were aliquoted and stored at -20°C.

### Western blotting

Total cell lysate was prepared by lysing the cells in RIPA lysis buffer (150 mM NaCl, 0.1% SDS, 0.5% Sodium Deoxycholate, 1% NP-40, and 50 mM Tris-Cl, pH 7.5) supplemented with the Protease Inhibitors Cocktail Set III and 1 mM PMSF at a density of 1 x 10^7^ cells/ml buffer. The cell suspension was agitated for 30 min and then centrifuged at 12,000 rpm for 20 min at 4°C. Then the supernatant was transferred to a new tube and stored at -20°C. The protein concentration was measured using DC protein assay kit (Bio-Rad, CA, USA). Next, 50 μg of protein from the cell lysates were separated by 12% SDS-polyacrylamide gel electrophoresis (SDS-PAGE) and electrotransferred onto nitrocellulose membranes (Bio-Rad, CA, USA). The membrane was blocked with 5% non-fat milk (Nestle, Switzerland) in PBS with 0.05% tween-20. This was followed by incubation overnight with a diluted solution of primary antibody. This was followed by incubation at room temperature with HRP-conjugated antibody (anti-mouse IgG or anti-rabbit IgG) (Cell Signaling Technology Beverly, MA) for 1 h. At least two independent experiments were performed. The blots were visualized by enhanced chemiluminescence (ECL) using the Western Blotting Luminol Reagent (GE Healthcare, UK). Images developed were captured with an LAS-4000 gel documentation system (Fuji Film, Tokyo, Japan).

### Statistical analysis

Statistical analyses were performed by Student′s t-test using the Origin 8.5 software (Originlab Corporation, Northampton, MA, USA) and Microsoft Excel program. All numerical data were presented as mean ± SD from at least 3 independent experiments. P*-*values of less than 0.05 were considered as significant.

## Results

### Effect of Andro combined with different flavonoids on HeLa cells

A screening study was done to test the combination effects of Andro with different flavonoids (Taxi, silibinin, quercetin, apigenin and luteolin) (data not shown). The combination effect of Andro with Taxi on growth inhibition of HeLa cells was synergistic at all tested combination ratios. Therefore we decided to study further and in more details the synergistic effects of this combination and to investigate the mechanism involved.

### Taxi synergized the antiproliferative effect of Andro on HeLa cells

**Fig 1:**
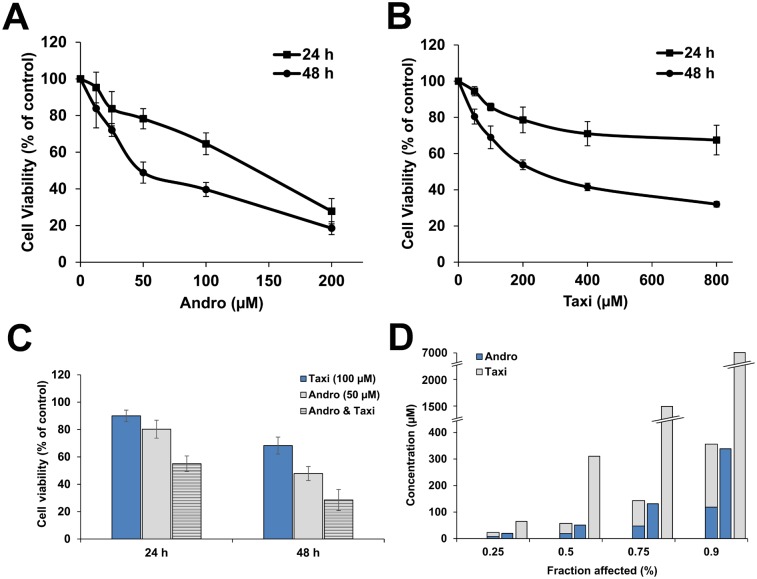
Effect of Andro and/or Taxi on the proliferation of HeLa cells. The cells were treated with different concentrations of Andro (A) or Taxi (B) alone or a combination of 50 μM Andro and 100 μM Taxi (C) for 24 or 48 h and then the cell viability was tested using the MTT assay. Values from each time point were then compared to the control values and expressed as mean ± SD of three independent experiments. (D) A graph representing the decrease in the total concentration of Andro and Taxi when used in combination compared to the single doses of Andro or Taxi.

Andro and Taxi inhibited the proliferation of HeLa cells in a dose- and time-dependent manner (Fig 1A and 1B) but, Taxi effect was much lower than Andro. The calculated IC_50_ of Andro and Taxi were 123 μM and 2 mM (24 h) while 51 μM and 310.5 μM (48 h) respectively. The combination of 50 μM Andro with 100 μM Taxi decreased the viability of cells from 80.3% to 55.1% (Combination index [CI]≈0.5) and from 47.9% to 28.5% (CI≈0.43) after treatment for 24 and 48 h respectively (Fig 1C). The dose reduction index (DRI) for the combination of Andro and Taxi which affected 75% of the cells after 48h treatment was 2.7 (the dose of Andro was reduced from ~135 to 50 μM) and 15.5 (the dose of Taxi was reduced from ~1.5 mM to 100 μM) respectively (Fig 1D).

The total combination concentration of Andro and Taxi at the different combination ratios 1:1, 1:2 and 1:4 which affected 75% of the cells were 127, 143.1 and 263.4μM with CI values 0.53, 0.43 and 0.53 respectively. Therefore, we chose the combination ratio of 1:2 which gave the best CI value. The concentrations of Andro and Taxi used to study further the effects of the combination were 50 μM and 100 μM respectively for the subsequent experiments.

### Taxi enhanced Andro-induced apoptosis in HeLa cells

**Fig 2:**
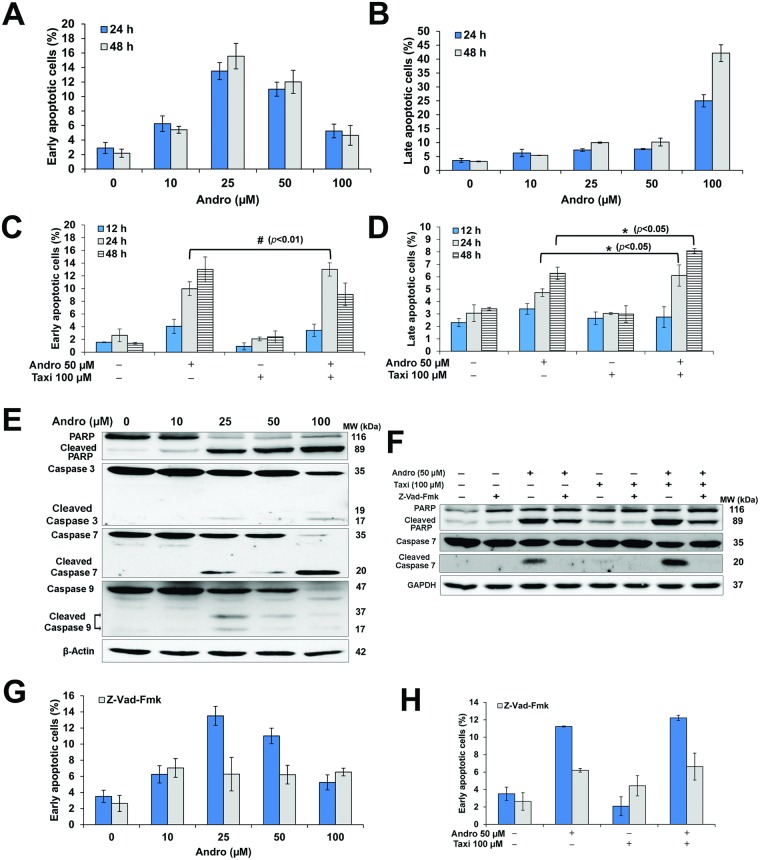
Effect of different concentrations of Andro and/or Taxi on Apoptosis in HeLa cells. (A, B, C & D) The mean percentages of early apoptotic; late apoptotic and dead cells treated with Andro and/or Taxi for specified time. The cells were treated as indicated, then collected, stained with Yo-Pro-1 and PI and analyzed using the flow cytometer. Results are represented as mean ± SD (n = 3). (A & B) The cells were treated with different concentrations of Andro for 24 or 48 h. (C & D) The cells were treated with Andro and/or Taxi for 12, 24 or 48 h. (E & F) Cells treated with Andro and/or Taxi for 24 h, then 50 μg of the protein lysate were resolved by electrophoresis and then detection of the different apoptotic proteins was done by western blotting. β-Actin and GAPDH were used to ensure equal protein loading. (E) Effect of different concentrations of Andro for 24 h on the expression and cleavage of caspases and PARP. (F) Effect of 50 μM Andro and/or 100 μM Taxi alone or in the presence of Z-Vad-Fmk for 24 h on the expression and cleavage of caspases and PARP. (G & H) The effect of the pan-caspase inhibitor Z-Vad-Fmk on apoptosis in HeLa cells treated with Andro and/or Taxi. Cells were pretreated for 1 hour with Z-Vad-Fmk (10 μM) and then with Andro and/or Taxi for 24 h. The cells were then collected and stained with Yo-Pro-1 and PI and analyzed using the flow cytometer. Results are represented as mean ± SD (n = 3). (G) Effect of Z-Vad-Fmk on apoptosis in cells treated with different concentrations of Andro for 24 h. (H) Effect of Z-Vad-Fmk on apoptosis in cells treated with 50 μM Andro and/or 100 μM for 24 h. * or # indicates significantly different from the control (*, *p* < 0.05; #, *p* < 0.01).

HeLa cells treated with Andro alone or Andro combined with Taxi showed features of apoptosis including nuclear condensation and membrane blebbing (S1 Fig). Andro-induced apoptosis in HeLa cells after treatment for 24 h with an increase in the mean percentage of early apoptotic cells at 25 μM and a decrease at concentrations 50 μM and 100 μM due to the increase in late apoptotic cells (Fig 2A and 2B). Early and late apoptosis were increased in combined-treatment compared to treatment with Andro alone (Fig 2C and 2D). The involvement of caspases in Andro-induced apoptosis was then examined. Andro caused an increase in the cleaved-PARP and other caspases as the concentration increased from 0–100 μM (Figs 2E and S3). Cells treated with 50 μM Andro and 100 μM Taxi for 24 h, increased the cleaved-PARP (~1.8-folds) and the cleaved-caspase-7 (~3-folds) compared to treatment with Andro alone (Figs 2F and S3A–S3C). The pan-caspase inhibitor, Z-Vad-Fmk, didn’t completely inhibit the apoptosis induced by Andro and/or Taxi (Fig 2G and 2H). The pretreatment with Z-Vad-Fmk attenuated completely caspase-7 cleavage and partially PARP cleavage in cells treated with 50 μM Andro and/or 100 μM Taxi (Figs 2F and S3E and S3F). Accordingly, these results suggest that the apoptosis induced by Andro alone or Andro combined with Taxi is partly mediated by caspase-dependent pathway and that the caspase-independent pathway may be also involved in the death process.

### Andro induced autophagy in HeLa cells while Taxi attenuated it

**Fig 3:**
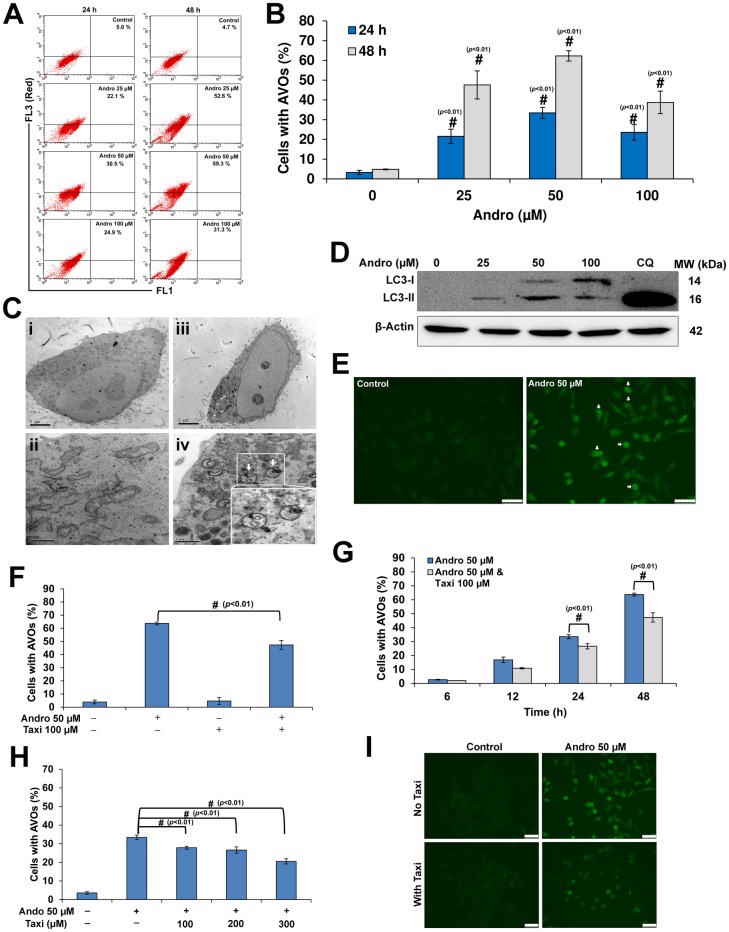
Andro induced autophagy in HeLa cells while Taxi diminished it. (A) A representative flow cytometric dot plots showing the increase in the AVOs percentage in HeLa cells treated with different concentration of Andro for 24 and 48 h and stained with acridine orange. (B) The percentage of cells with AVOs as determined by flow cytometer after cells were treated for 24 or 48 h with different concentrations of Andro and stained using acridine orange. Results are represented as mean ± SD (n = 3). (C) Representative transmission electron micrographs showing the AVOs in HeLa cells treated 50 μM Andro for 24 h (Ciii & Civ) compared to cells treated with DMSO (controls) (Ci & Cii). White arrows indicate the AVOs. Upper panels, scale bar = 5 μM, lower panels, scale bar = 1 μM. (D) Western blotting analysis of LC3-II protein levels in protein lysates from HeLa cells treated with different concentrations of Andro for 24 h. Chloroquine (5 μM) was used as a positive control and β-Actin was used to ensure equal protein loading. (E) Immunofluorescence micrographs showing punctuate green fluorescence patterns in HeLa cells treated with 50 μM Andro for 24 h compared to the control. # indicates significantly different from the control (#, *p*< 0.01), scale bar = 75 μm. (F, G & H) The cells were treated as indicated for the specified time, stained with acridine orange and analyzed using the flow cytometer. (F) The percentage of HeLa cells with AVOs after treatment with 50 μM Andro and/or 100 μM Taxi for 48 h. (G) The percentage of HeLa cells with AVOs after treatment with 50 μM Andro alone or combined with 100 μM Taxi at different time points. (H) The percentage of HeLa cells with AVOs after treatment with 50 μM alone or in combination with different concentrations of Taxi for 24 h. Results are represented as mean ± SD (n = 3). (I) Immunofluorescence micrographs showing the punctuate green fluorescence patterns in HeLa cells treated with 50 μM Andro and/or 100 μM Taxi for 24 h compared to the control. # indicates significantly different (#, *p*< 0.01), scale bar = 75 μm.

Autophagy may act either as prosurvival or prodeath signal [37–41]. Different techniques were used to detect the Andro-induced autophagy in HeLa cells as indicated in materials and methods. Firstly, Andro increased the mean percentage of AVOs in a time- and dose-dependent manner (Fig 3A and 3B). Secondly, TEM revealed the appearance of double-membranous cytoplasmic vacuoles (white arrows) with some showing entrapped organelles after Andro treatment for 24 h (Fig 3C). Thirdly, Andro increased LC3-II form detected using western blotting (Figs 3D and S3G). Finally, punctuate green fluorescence patterns appeared (white arrows) in cells treated with Andro (Fig 3E). On the other hand, the combination of Andro with Taxi decreased autophagy significantly in a time- and dose-dependent manner (Fig 3F–3H). This was further confirmed by the decrease in punctuate green fluorescence patterns in cells (Fig 3I).

### Andro induced-protective ROS-dependent autophagy

**Fig 4:**
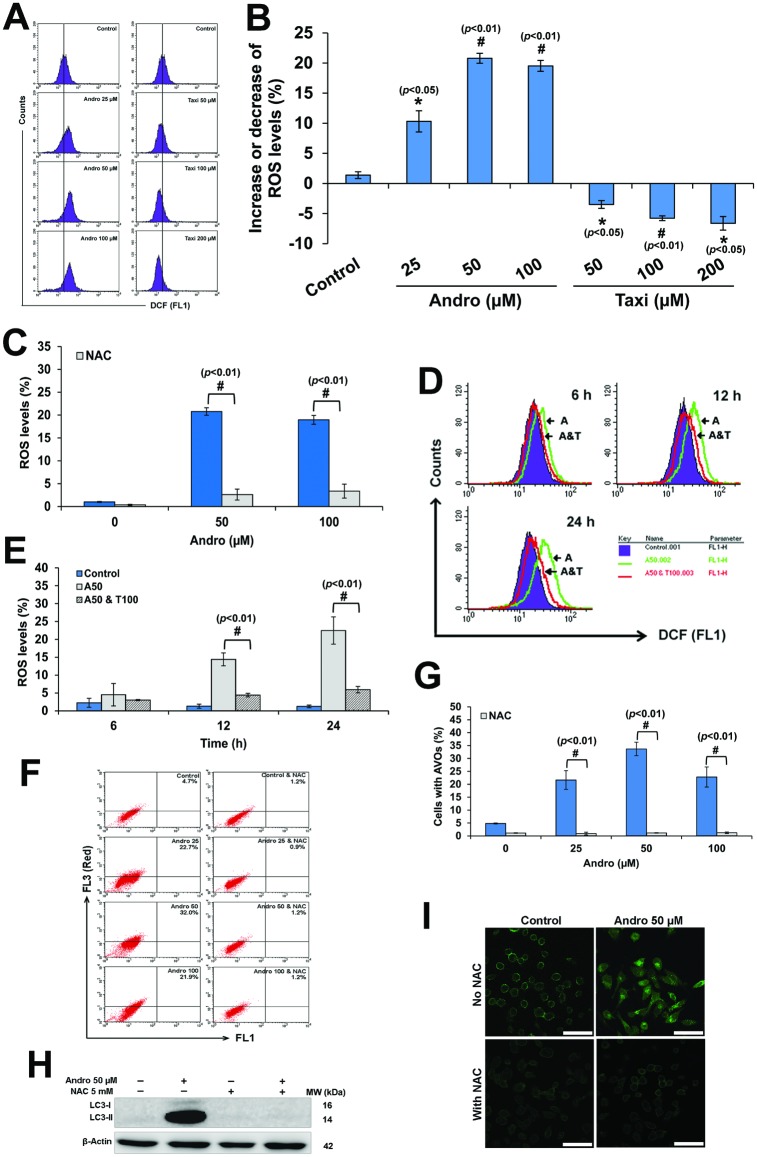
Andro induced ROS-dependent autophagy while Taxi attenuated it. Cells were treated as indicated, stained with DCF-DA and then analyzed using the flow cytometer. (A) A representative histograms showing the increase in ROS in HeLa cells treated with Andro (25, 50 or 100 μM) while the decrease in ROS in those treated with Taxi (50, 100 or 200μM) for 24h. (B) The percentage of cells with higher or lower levels of ROS compared to the control in HeLa cells treated with different concentrations of Andro or Taxi for 24 h. (C) The percentage of ROS levels in cells treated with different concentrations of Andro with or without pretreatment with NAC. The cells were either pretreated or not with NAC (5 mM) for 1 h, and then different concentrations of Andro were added and the cells were incubated for another 24 h. (D) A representative overlay histograms of ROS levels in HeLa cells treated with 50 μM Andro and/or 100 μM Taxi for different time points. (E) The percentage of ROS levels in HeLa cells treated with 50 μM Andro and or 100 μM Taxi for different time points. (B, C & E) Results are represented as mean ± SD (n = 3). (F) A representative dot plot showing the effect of different concentrations of Andro on autophagy in HeLa cells with or without 1 h pretreatment with NAC (5 mM). (G) The percentage of AVOs in HeLa cells pretreated with NAC (5 mM) for 1 h, then, treated with different concentrations of Andro as indicated for 24 h. Results are represented as mean ± SD (n = 3). (H) Western blotting analysis of LC3-II protein levels in protein lysates from HeLa cells treated with 50 μM Andro for 24 h with or without 1 h pretreatment with NAC (5 mM). β-Actin was used to ensure equal protein loading. (I) Immunofluorescence micrographs showing the punctuate green fluorescence patterns in HeLa cells treated with 50 μM Andro for 24 h with or without 1 h pretreatment with NAC (5 mM). # or * indicates significantly different (#, *p*< 0.01; (*, *p*<0.05), scale bar = 75 μm.

Andro induced ROS generation in HeLa cells after 24 h treatment while Taxi attenuated it (Fig 4A and 4B). The addition of NAC (5 mM) to the cells treated with Andro attenuated the ROS generation and returned the ROS levels roughly to the basal control level (Fig 4C). On the other hand, Taxi attenuated the Andro-induced ROS generation at different time points (Fig 4D and 4E). The addition of NAC to the cells treated with different concentrations of Andro abolished almost completely autophagy represented by the decrease in AVOs (Fig 4F and 4G). This was further confirmed by the abolished conversion of LC3-I to LC3-II and the disappearance of green punctate patterns (Figs 4H and 4I and S3H).

**Fig 5:**
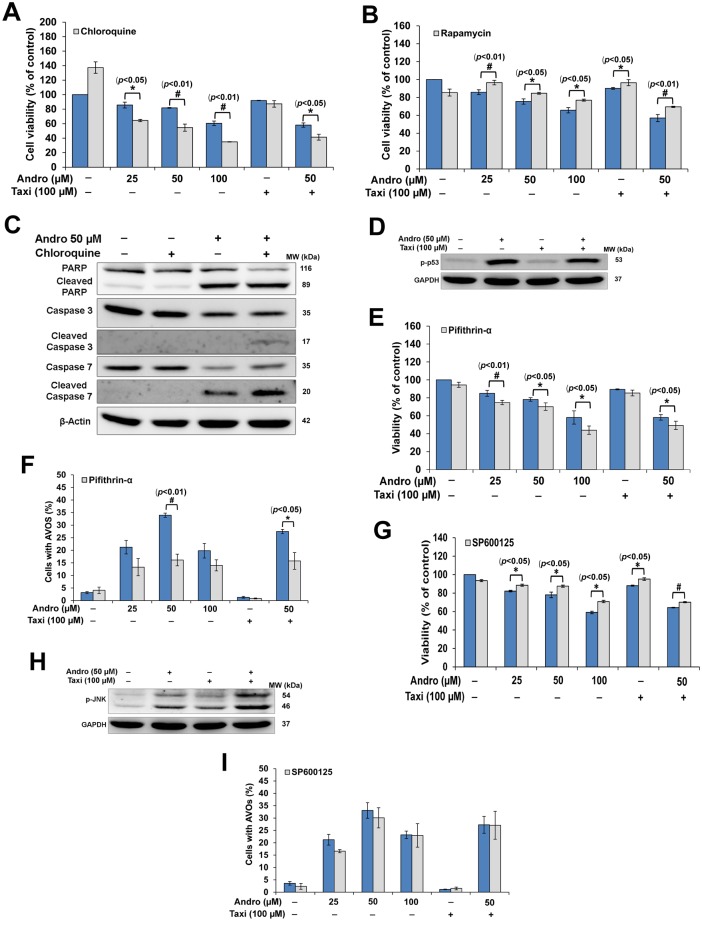
Inhibition of protective autophagy augmented apoptosis in HeLa cells via reduction of p- p53. (A & B) The cells were treated with different concentrations of Andro, 100 μM Taxi or 50 μM Andro combined with 100 μM Taxi for 24 h with or without 1 h pretreatment with CQ (5 μM) or Rapamycin (0.5 μM). The cell viability was tested using the MTT assay. Values from each treatment were compared to the control values and expressed as mean ± SD of three independent experiments. (C) Apoptosis as illustrated by cleavage of PARP and caspases 3 and 7 following 24 h Andro treated Hela cells with or without 1 h pretreatment with CQ (5μM). (D) Effect of Andro and/or Taxi on p-p53 protein levels. GAPDH was used to ensure equal protein loading. (E) Effect of Andro and/or Taxi in the presence or absence of PFT-α on viability of HeLa. (F) Effect of Andro and/or Taxi in the presence or absence of PFT-α on autophagy of HeLa. (G) Western blotting analysis of p-JNK protein levels from HeLa cells treated with 50 μM Andro and or 100μM Taxi for 24 h. (H) Effect of Andro and/or Taxi in the presence or absence of SP600125 (5 μM) on viability of HeLa after 24 h. (I) The percentage of HeLa cells with AVOs after treatment with different concentrations of Andro or with Andro combined with Taxi for 24 h with or without 1 h pretreatment with SP600125 (5 μM). Results are represented as mean ± SD (n = 3). * or # indicates significantly different (*, *p*< 0.05; #, *p*< 0.01).

Subsequently, we investigated whether Andro-induced autophagy is a prosurvival or a proapoptotic signal. MTT results show that the pretreatment of cells with chloroquine (5 μM) followed with Andro alone or in combination with Taxi for 24 h decreased the cell viability (Fig 5A) meanwhile viability was increased when cells were pretreated with rapamycin (Fig 5B). Taken together, the results confirm that Andro-induced autophagy is ROS-dependent and protective in HeLa cells.

### Inhibition of autophagy increased the apoptosis in HeLa cells via the reduction of p-p53

Hela cells treated with Andro and CQ (5 μM) increased the cleaved-PARP (1.25-folds), cleaved-caspases-3 (2-folds) and cleaved-caspase-7 (1.5-folds) compared to treatment with Andro alone (Figs 5C and S3I–S3K). Whereas, the concentration of p-p53 increased to 9.1-folds (Andro-treated) and decreased to 6.8-folds (combined-treatment) compared to the control (Figs 5D and S3L). Recently, p53 was found to be involved in the regulation of autophagy. Therefore, we asked if p53 is involved in the combined-treatment reduced autophagy or not. PFT-α was used, specifically inhibits p53, to test the effect of inhibition of p53 on the viability and autophagy in HeLa cells. Cells pretreated with 30 μM PFT-α and then with Andro and/or Taxi for 24 h caused a significant decrease in their viability (Fig 5E). Meanwhile, cells pretreated with PFT-α, and then with Andro and/or Taxi caused a decrease in the mean AVOs percentage which was significant at 50 μM Andro and in the combination treatment (Fig 5F). These results confirm that p53 has a role in Andro-induced autophagy and also confirm that the reduction in autophagy due to the combined-treatment is caused by the reduction in p53.

### JNK has a role in Andro-induced cell death but not in autophagy

Treatment of cells with Andro alone or combined with Taxi increased the p-JNK by 3.9-folds and 14.7-folds respectively compared to the control (Figs 5H and S3M). The use of SP600125 (JNK inhibitor) increased the viability of HeLa cells significantly but did not rescue the cells completely from the effect of Andro alone or in combination (Fig 5I). On the other hand, SP600125 did not have any significant effect on autophagy in cells treated with Andro and/or Taxi (Fig 5I). Accordingly, the results show that Andro combined with Taxi increased the concentration of p-JNK which may have a role in the induced cell death but is not involved in the induction of autophagy.

### Taxi enhanced the MMP loss induced by Andro in HeLa cells while JNK inhibitor reduced it

**Fig 6:**
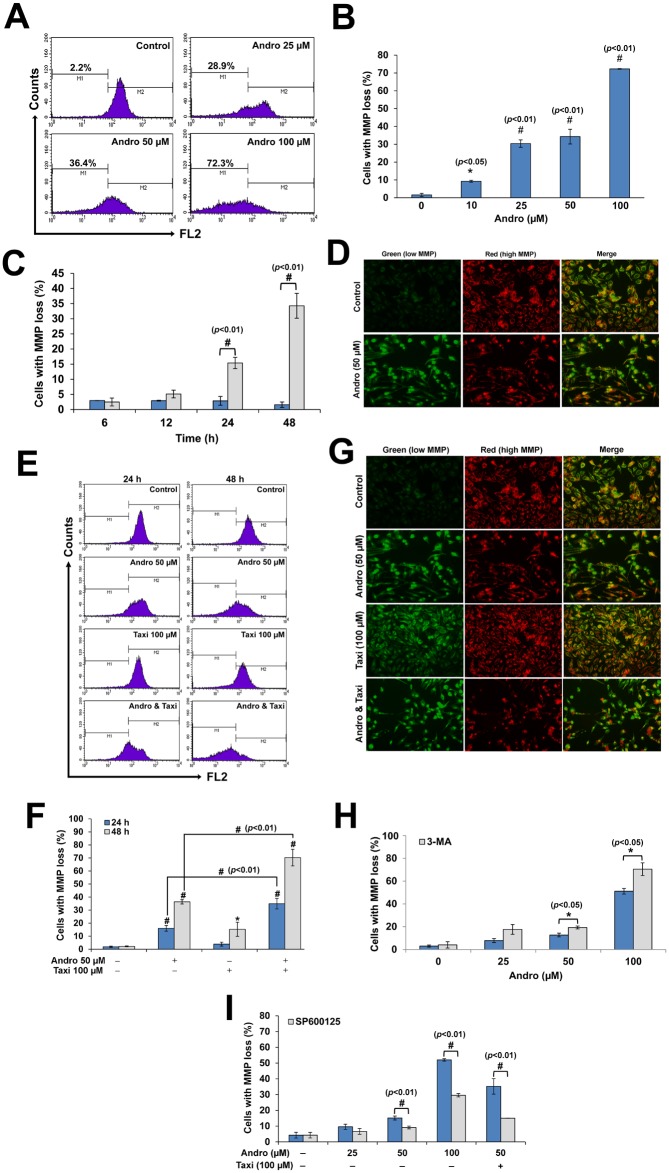
The effect of Andro alone or in combination with Taxi on MMP loss in HeLa cells. (A) A representative flow cytometric histograms showing the loss in MMP (decrease in FL2 fluorescence) in HeLa cells treated with different concentrations of Andro for 48 h and stained with JC-1. (B) The percentage of cells with MMP loss in HeLa cells treated with different concentrations of Andro for 48 h. (C) The percentage of cells with MMP loss in HeLa cells treated with 50 μM Andro for different time points compared to the control. Results are represented as mean ± SD (n = 3). (D) Immunofluorescence micrographs of HeLa cells stained with JC-1 showing the increase in the J-aggregates form (red) with low monomeric form (green) in the control, while a decrease in the J-aggregates form (red) and increase in the monomeric forms (green) in cells treated with 50 μM Andro, scale bar = 50 μm. (E) A representative flow cytometric histograms showing the loss in MMP (decrease in FL2 fluorescence) in HeLa cells treated with 50 μM Andro and/or 100 μM Taxi for 24 or 48 h and stained with JC-1. (F) The percentage of cells with MMP loss in HeLa cells treated with 50 μM Andro and/or 100 μM Taxi for 24 or 48 h and stained with JC-1. (G) Immunofluorescence micrographs of HeLa cells stained with JC-1 showing the increase in the J-aggregates form (red) with low monomeric form (green) in the control, while a decrease in the J-aggregates form (red) and increase in the monomeric forms (green) in cells treated with 50 μM Andro and/or 100 μM Taxi, scale bar = 50 μm. (H) The percentage of cells with MMP loss in HeLa cells treated with different concentrations of Andro for 24 h with or without 1 h pretreatment with 3-MA (5 mM). (I) Effect of SP600125 on the MMP on HeLa cells treated with Andro or Andro and Taxi for 24 h. Results are represented as mean ± SD (n = 3). # indicates significantly different (*, *p*< 0.05; #, *p*< 0.01).

Andro caused a dose- and time-dependent MMP loss due to the increase in MOMP (Fig 6A–6C). HeLa cells treated with Andro combined with Taxi further increased the loss of MMP (Fig 6E and 6F). This was further confirmed by the fluorescence microscope where the JC-1 probe was used (Fig 6D and 6G). This trend was also found when HeLa cells were pretreated with 3-MA and then with Andro (Fig 6H). On the other hand, treatment of HeLa cells with Andro alone or in combination with Taxi in the presence of SP600125 caused a decrease in the MMP loss. This further confirms that the increase in the p-JNK is involved in the increase loss of MMP (Fig 6I).

### Effect of Andro and/or Taxi on AIF, cytochrome *c* and pro-apoptosis proteins release

**Fig 7:**
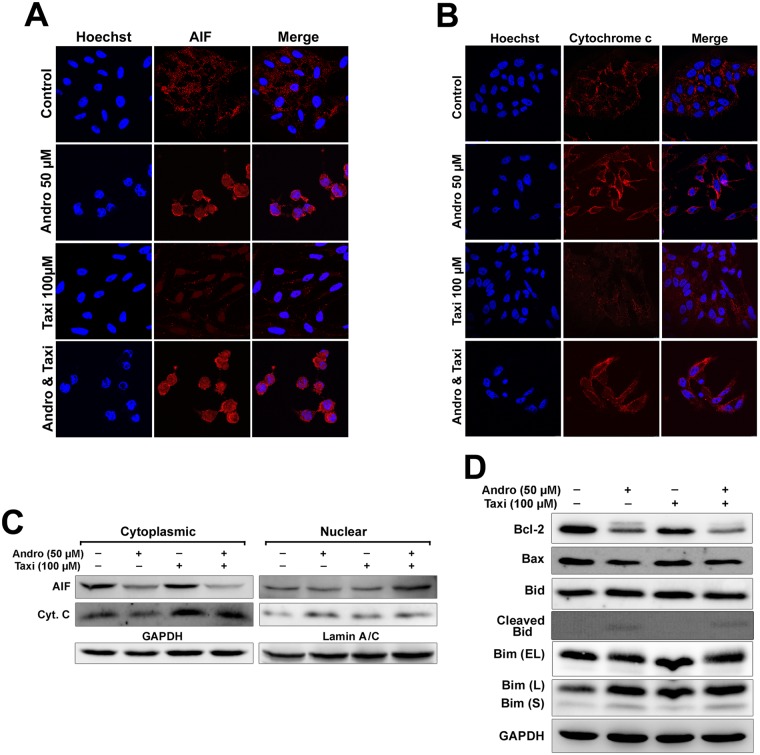
Role of AIF, Cytochrome *c* and proapoptotic factors in the cell death induced by Andro and/or Taxi on Hela cells. (A & B) Effect of Andro and/or Taxi on translocation of AIF and cytochrome *c* to the nucleus in HeLa cells. Cells were seeded on sterile coverslips, treated with 50 μM Andro and/or 100 μM Taxi for 24 h, fixed and immunostained with anti-AIF or anti-cytochrome *c* antibody, CFL-conjugated secondary antibody, counterstained with Hoechst 33342 and then observed under the confocal microscope, scale bar = 20 μm. (C) Effect of Andro and/or Taxi on the expression of AIF and cytochrome *c* proteins. (D) Effect of Andro and/or Taxi on the expression of Bcl-2 and the proapoptotic proteins. GAPDH was used to ensure equal protein loading. Results are represented as mean ± SD (n = 3). # indicates significantly different (#, *p* < 0.01).

AIF was located in the cytoplasm in the control cells, while treatment of cells with Andro and/or Taxi caused AIF translocation to the nucleus (Fig 7A) which was 2-folds more in the combined-treatment compared to Andro alone. Whereas, cytochrome *c* translocation from the cytoplasm to the nucleus was minor (Fig 7A and 7B), the cytoplasmic cytochrome *c* concentration increased in the combination treatment compared to treatment with Andro alone (Figs 7C and S3N and S3O). The proapoptotic protein Bid was cleaved in cells treated with Andro alone or with Andro combined with Taxi. There was also an increase in Bim (L) and Bim (S) proteins concentration in cells treated with Andro (1.2- and 1.4-folds respectively) and when Andro combined with Taxi (1.6- and 1.7-folds respectively) compared to the control (Figs 7D and S3P–S3T).

## Discussion

The combination effect of Andro with Taxi on growth inhibition of HeLa cells was synergistic at all tested combination ratios contrast to the results of other flavonoids (silibinin, quercetin, apigenin & luteolin) which ranged from antagonism to synergism depending on the combination ratio and the concentrations used. In fact, the flavonoid concentration is an important factor in its mode of action, the effect it will exert on cells and also on its interaction with other compounds [42].

The combined-treatment of Andro with Taxi led to a higher early and late apoptosis compared to treatment with Andro alone (Fig 2C and 2D). However, the early apoptosis results were not compatible with the MTT antiproliferation results which suggested the involvement of caspase-independent cell death. This was confirmed by using Z-Vad-Fmk (Fig 2G and 2H) and western blotting (Fig 2F). It is widely accepted nowadays that the tumor response to therapy is a heterogeneous model where various modes of death combine to generate the overall tumor response [43]. The factors which determine the mechanisms of cell death include mechanism of action of the drug, dosing regimen used, and the genetic background of the cells within the tumor [43].

Recently, different studies have found diverse effects of autophagy on cancer cells, either survival or death, depending on the cancer and on the environment around the cancer cells [44]. Two studies have shown controversial results of Andro on autophagy induction in different cancer cell lines [33, 34]. Our results confirmed that Andro induced autophagy in Hela cells (Fig 3A–3E). On the other hand, the combination of 50 μM Andro with 100 μM Taxi caused a significant decrease in autophagy (Fig 3F–3I). Moreover, the decrease in autophagy in the combination treatment was not cell-type specific (S2 Fig). The results also showed that the induction of autophagy is ROS-dependent (Fig 4G–4I) which coincides with the results of different studies [10, 45]. Conversely, Taxi was shown to act as an antioxidant and decreased ROS production [46]. The combined-treatment of Andro and Taxi caused a decrease in ROS generation (Fig 4E) and at the same time reduced autophagy (Fig 3F–3I). Thus, the antioxidant effect of Taxi which caused the decrease in ROS generation caused a reduction of Andro-induced autophagy. In general, most of the dietary supplements have health-improving effects generally via strengthening the cellular antioxidant system in the body [47]. Those dietary supplements which also possess anticancer activity have more advantage and may give better results in combination chemotherapy [47]. In our study, Taxi worked as both antioxidant and anticancer agent against HeLa cells and these characteristics may be responsible for its ability to synergize the effects of Andro.

Our results showed that Andro induced protective- autophagy in which its inhibition decreased cell viability while increased apoptosis of HeLa cells (Fig 5A and 5C). These results further confirm that Taxi acted as an inhibitor of autophagy whenever combined with Andro and consequently, the increase in caspase-dependent apoptosis is attributed to this inhibition. Therefore, our results agree with many studies which demonstrated the autophagy-protective role in chemoresistant apoptosis [37–39, 48, 49] while disagree with others who indicated that cell death was due to autophagy and not apoptosis [50]. Many factors are involved in determining the destiny of the cells including the type of stimuli, nutrient availability and apoptotic status of cells [51]. Therefore, the determination of a drug’s effect on autophagy may be an important factor in knowing the major impediments to a successful cancer therapy.

At the molecular level, different molecules are involved in autophagy induction including p53 and JNK. In addition to the known functions of p53, the most important being cell cycle arrest and apoptosis, it was also recently found to be involved in the regulation of autophagy [52–54]. In the present study, we confirmed by using the p53 inhibitor, PFT-α, that p53 has a role in Andro-induced autophagy where the inhibition of p53 caused a decrease in autophagy. This agrees with many studies which showed that ROS and p53 are playing important roles in the induction of autophagy [41, 55]. Our results also confirm that the reduction in autophagy due to the combined-treatment is caused by the reduction in p53. This was further confirmed by the results of MTT which showed that the inhibition of p53, which caused reduction in autophagy, also caused a decrease in the viability of HeLa cells and further confirms that Andro-induced autophagy is protective.

On the other hand, JNK was shown to have a critical role in autophagy [55, 56]. Our results showed that Andro combined with Taxi increased the cytotoxic effect on HeLa cells through the activation of JNK where the inhibition of JNK by SP600125 increased the cell viability (Fig 5I). The results also showed that the activation of JNK is not involved in autophagy (Fig 5J). Our results disagree with those of Notte *et al*. who found that JNK promoted survival for the MDA-MB-231 breast cancer where the cells became resistant to taxol-induced apoptosis due to JNK activation while agree with them in that JNK plays no role in the induction of autophagy [57]. In contrast, our results agree with those of Shimizu *et al*. and He *et al*. who found that the activation of JNK is involved in the cell death and that the use of JNK inhibitor suppressed it [56, 58].

The phosphorylation and stabilization of p53 was reported to be regulated by JNK [59, 60]. In contrast, other reports indicated that the activation of JNK can reduce p53. In fact, our results showed that the activation of JNK reduced the p-p53. This agrees with Wang *et al*. results who reported that Short-chain fatty acid mixes sustained the activation JNK1 and downregulation of p53 [61]. Tafolla *et al*. also found that JNK1 and JNK2 are opposite regulators of p53 where JNK1 is a negative regulator while JNK2 is a positive regulator of p53 expression [62].

The involvement of mitochondrial-events in autophagy process was reported in many studies [33, 38]. The treatment of HeLa cells with Andro caused an increase in the MMP loss (Fig 6B) which was augmented by either the addition of Taxi (Fig 6F) or 3-MA (Fig 6H). Therefore, the decrease in autophagy augmented the loss of MMP. Similar results were shown by different groups and on different cancer cells where 3-MA increased the MMP loss when used with cisplatin, 5-FU [36] or with bortezomib [63]. The results showed that the treatment of HeLa cells with Andro and/or Taxi combined with SP600125 decreased the MMP loss. This further confirms that the increase in JNK-activation disrupted the mitochondria, increased the MMP loss, and increased the release of caspase-dependent and independent factors which caused cell death in HeLa cells. These results confirm further the important role of mitochondria in the cell death and further confirm the significance of autophagy in reducing the caspase-dependent and independent cell death by reducing the number of mitochondria by sequestration and destructing them in the autophagosomes.

The results also showed that AIF was translocated from the cytoplasm to the nucleus in the cells treated with Andro alone or Andro combined with Taxi, however the translocation was minor in the case of cytochrome *c* (Fig 7A and 7B). This was further confirmed by western blotting (Fig 7C). In the case of cytochrome *c*, there were higher levels of cytoplasmic cytochrome *c* in the cells treated with Andro combined with Taxi compared to treatment with Andro alone. The results confirm that there was another mechanism of cell death together with the caspase-dependent mechanism which is caspase-independent and related to the release of AIF and its translocation to the nucleus. These results coincide with many reports which also indicated that AIF is involved in caspase-independent cell death [38, 64].

**Fig 8:**
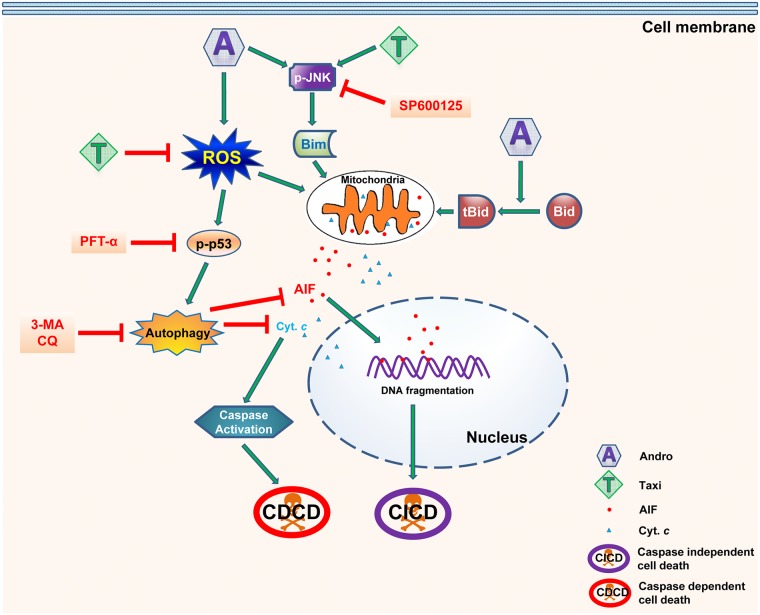
The proposed mechanism for Andro and Taxi induced-cell death in HeLa cells. Andro caused a genotoxic stress in HeLa cells which was associated with increase in ROS, MOMP, activation of JNK, p-p53 and apoptosis. Andro also induced autophagy in HeLa cells which was cytoprotective. The combination of Andro with Taxi decreased autophagy by decreasing ROS and p-p53. The use of the p-p53 inhibitor PFT-α also caused attenuation of autophagy. The combination treatment also increased the activation of JNK, the levels of tBid and Bim which increased further the MOMP and the release of AIF and cytochrome *c* and subsequently caused caspase-3independent and dependent cell death. The use of the JNK inhibitor SP600125 reduced the MMP loss and increased the viability of HeLa cells taxi.

In conclusion, the effects of Andro alone or in combination with Taxi on HeLa cells were investigated. Taxi, which has a low cytotoxic effect, synergized the cytotoxic effect of Andro by attenuating ROS and autophagy, increasing MMP loss, the release of AIF and cytochrome *c* and the caspase-dependent and independent cell death whenever they are in combined use. Based on the results, a proposed pathway for cell death was formulated (Fig 8). To our knowledge, the present study has shown for the first time that Andro can induce protective autophagy in HeLa cells and that the autophagy reduced by Taxi can improve the cytotoxic effect of Andro. This can open the door for further investigation of the autophagy inhibitory effects of other flavonoids in combination with other chemotherapeutic drugs which are known to induce autophagy-induced chemoresistance. These results added more knowledge in understanding the effects of Andro on cancer cells which may lead to the improvement of its cytotoxic effects and future use in the treatment of cancer.

## Supporting information

S1 Fig Morphological changes in HeLa cells treated with Andro and/or Taxi for 48 h. (DOCX)

S2 Fig Andro induces autophagy in MCF-7 cells while Taxi reduces it. (DOCX)

S3 Fig Densitometric analysis of western blots. (DOCX)

## Abbreviations

DCF-DA: 2′,7′-dichorofluorescein diacetate
MTT: 3-(4, 5-dimethylthiazol-2-yl)-2, 5-diphenyl-tetrazolium bromide
3-MA: 3-Methyladenine
AVOs: Acidic Vesicular Organelles
AIF: Apoptosis-inducing factor
Andro: Andrographolide
Bcl-2: B cell lymphoma gene 2
Bid: BH3-interacting DD agonist
Bim: Bcl-2 interacting mediator of cell death
CI: Combination index
CQ: Chloroquine
DMSO: Dimethyl sulfoxide
DRI: Dose Reduction Index
GAPDH: Glyceraldehyde-3-phosphate dehydrogenase
JNK: c-Jun N-terminal kinases
LC3: Microtubule-associated protein-1 light chain 3
MMP: mitochondrial membrane potential
MOMP: mitochondrial outer-membrane permeabilization
NAC: N*-*acetylcysteine
PARP: Poly (ADP-ribose) Polymerase
PBS: Phosphate buffer saline
PFT- α: Pifithrin-α
PI: Propidium iodide
ROS: Reactive oxygen species
SDS: Sodium Dodecyl Sulfate
SDS-PAGE: SDS-polyacrylamide gel electrophoresis
Taxi: Taxifolin
TEM: Transmission Electron Microscopy
