# Supplementary material for: Taxifolin synergizes Andrographolide-induced cell death by attenuation of autophagy and augmentation of caspase dependent and independent cell death in HeLa cells
Source: PLoS One. 2017 Feb 9;12(2):e0171325. doi: 10.1371/journal.pone.0171325 (PMC5300218; doi:10.1371/journal.pone.0171325)
Supplement: S1 Fig — (DOCX) [file pone.0171325.s001.docx]

# S1 Figure


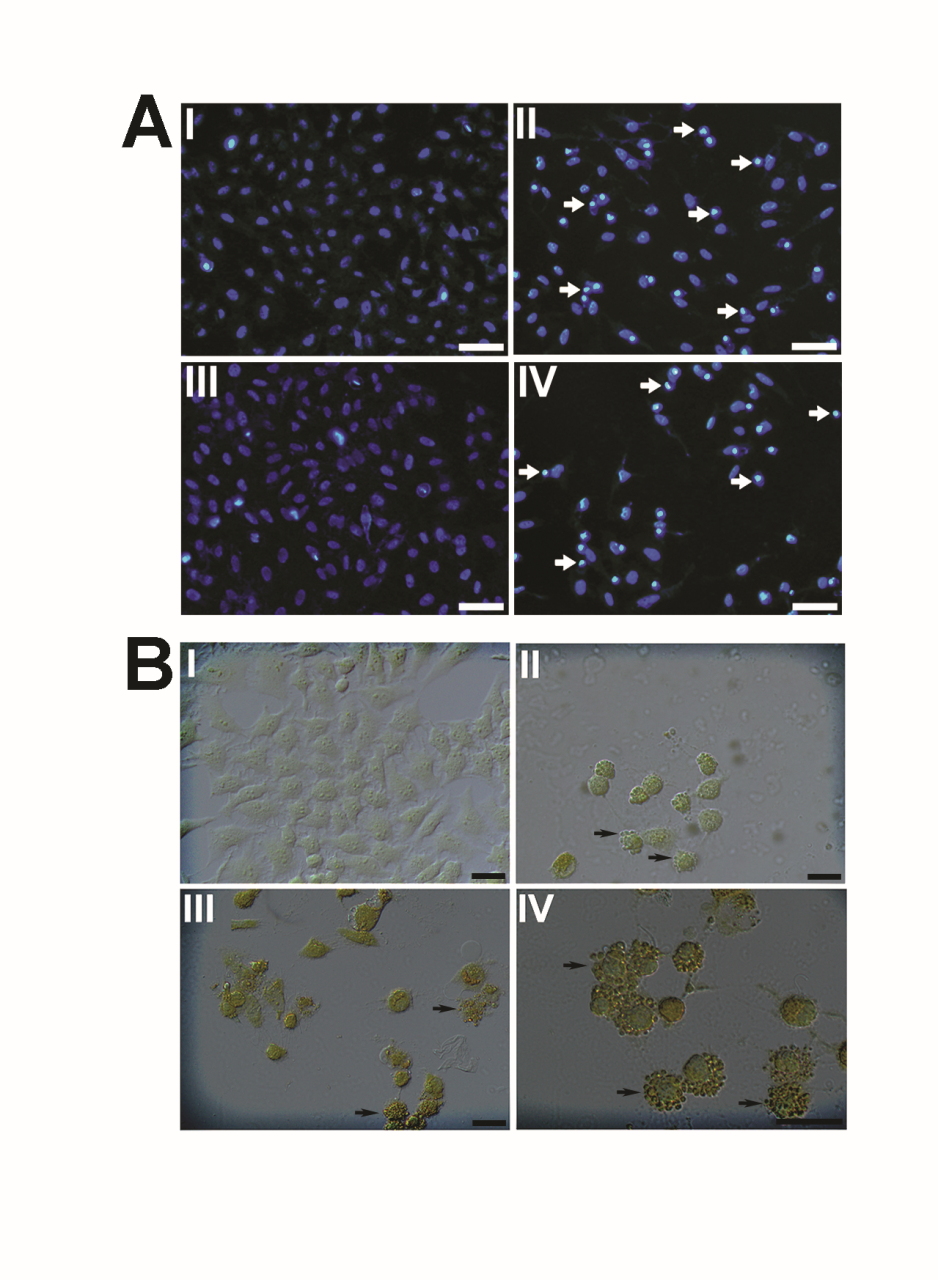


S1 Fig. Morphological changes in HeLa cells treated with Andro and/or Taxi for 48 h. Cells were seeded on coverslips as described in materials and methods and after 24 treated with 50 µM Andro and/or 100 µM Taxi for 48 h. Cells were then stained using Hoechst 33342, mounted on a slide using mounting medium and observed under the microscope. (A) the fluorescence microscope representing the nuclear condensation. (AI) control; (AII) Andro 50 µM; (AIII) Taxi 100 µM and (IV) Andro 50 µM with Taxi 100 µM. Scale bar = 100 µm. (B) using differential interference contrast (DIC) microscopy representing the membrane blebbing. (BI) Control; (BII) Andro 50 µM; (BIII) Andro 50 µM & Taxi 100 µM; and (BIV) Andro 50 µM & Taxi 100 µM (magnified 2X), scale bar = 25 µm. White arrows indicate the nuclear condensation while black arrows indicate membrane blebbing.
