# Supplementary material for: Taxifolin synergizes Andrographolide-induced cell death by attenuation of autophagy and augmentation of caspase dependent and independent cell death in HeLa cells
Source: PLoS One. 2017 Feb 9;12(2):e0171325. doi: 10.1371/journal.pone.0171325 (PMC5300218; doi:10.1371/journal.pone.0171325)
Supplement: S2 Fig — (DOCX) [file pone.0171325.s002.docx]

# S2 Figure


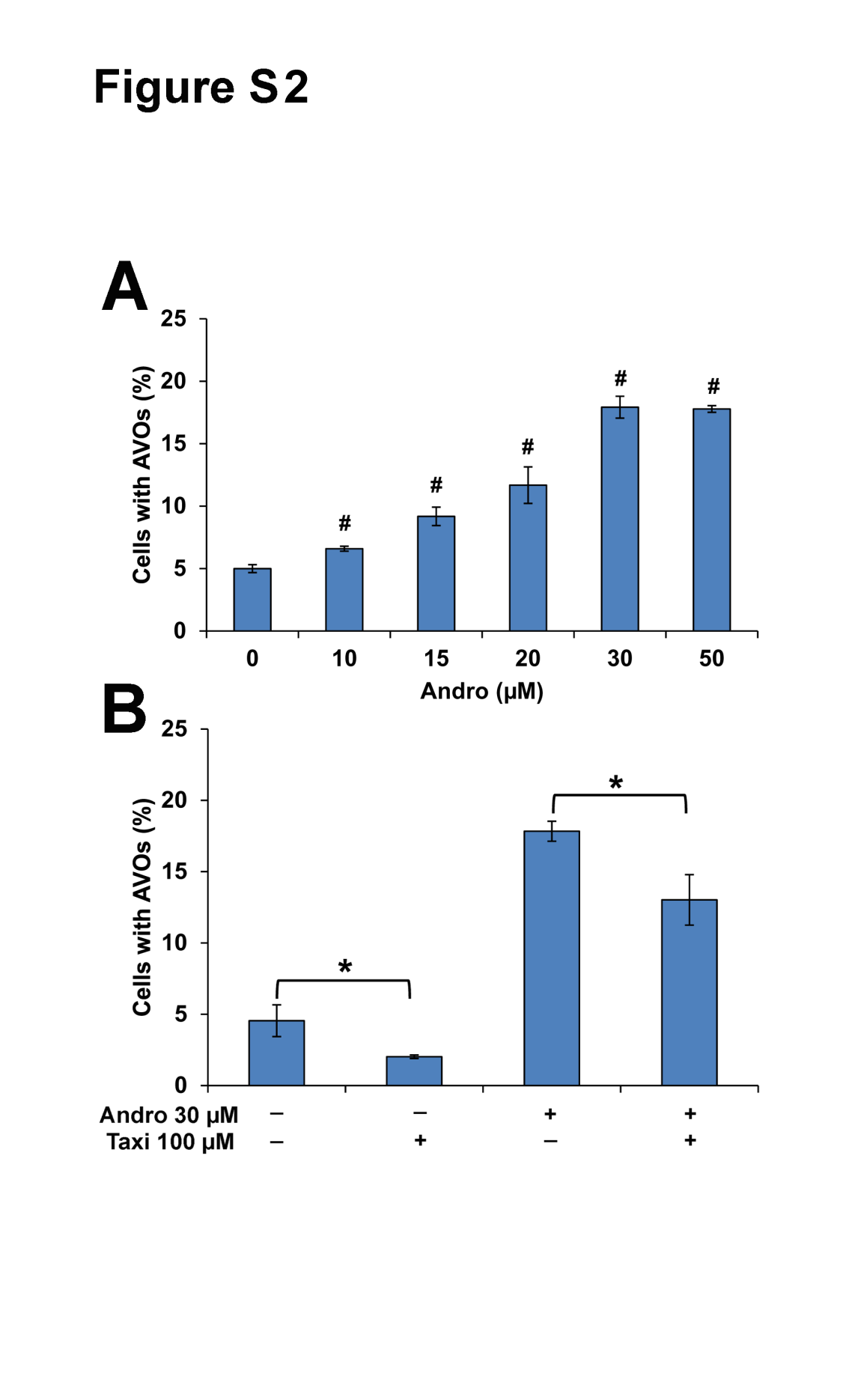


## Figure S2. Andro induces autophagy in MCF-7 cells while Taxi reduces it.

(A) The percentage of cells with AVOs was determined using the flow cytometer after cells were treated for 48 h with different concentrations of Andro (10, 15, 20, 30 or 50) and stained using acridine orange. (B) The percentage of cells with AVOs was determined using the flow cytometer after cells were treated for 48 h with different concentrations of 30 µM Andro and/or 100 µM Taxi and stained using acridine orange. Results are represented as mean ± SD (n = 3). * or # indicates significantly different (*, *p*< 0.05; #, *p*< 0.01).
