# Supplementary figures and images for: Taxifolin synergizes Andrographolide-induced cell death by attenuation of autophagy and augmentation of caspase dependent and independent cell death in HeLa cells
Source: PLoS One. 2017 Feb 9;12(2):e0171325. doi: 10.1371/journal.pone.0171325 (PMC5300218; doi:10.1371/journal.pone.0171325)

S3 Figure


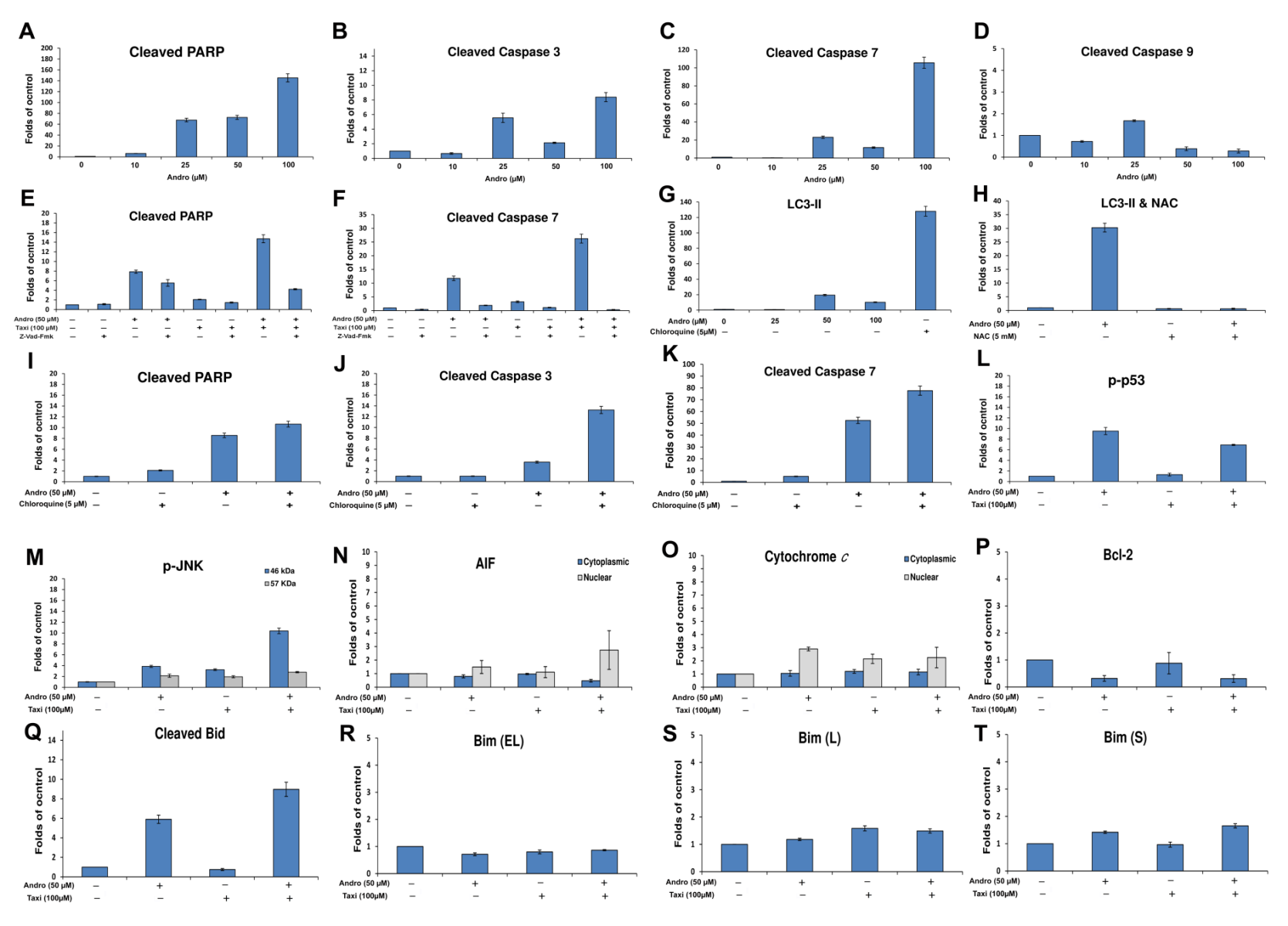


S3 Fig. Densitometric analysis of western blots.

Supplement: S3 Fig — (DOCX) [file pone.0171325.s003.Docx]
